# Privacy-preserving local analysis of digital trace data: A proof-of-concept

**DOI:** 10.1016/j.patter.2022.100444

**Published:** 2022-02-08

**Authors:** Laura Boeschoten, Adriënne Mendrik, Emiel van der Veen, Jeroen Vloothuis, Haili Hu, Roos Voorvaart, Daniel L. Oberski

**Affiliations:** 1 Department of Methodology and Statistics, Utrecht University, Padualaan 14, 3584 CH Utrecht, the Netherlands; 2 Centraal Bureau voor de Statistiek, Henri Faasdreef 312, 2492 JP The Hague, the Netherlands; 3 Eyra Leap B.V., Saturnusstraat 14, 2516 AH The Hague, the Netherlands; 4 Research and Data Management Services, Utrecht University, Heidelberglaan 8, 3584 CS Utrecht, the Netherlands; 5 Julius Center, University Medical Center Utrecht, Universiteitsweg 100, 3584 CG Utrecht, the Netherlands

**Keywords:** data donation, digital trace data, privacy, local processing, data extraction, software, proof-of-concept

## Abstract

We present PORT, a software platform for local data extraction and analysis of digital trace data. While digital trace data hold huge potential for social-scientific discovery, their most useful parts have been unattainable for scientists because of privacy concerns and prohibitive access to application programming interfaces. Recently, a workflow was introduced allowing citizens to donate their digital traces to scientists. In this workflow, citizens’ digital traces are processed locally on their machines before providing informed consent to share a subset of the data with researchers. In this paper, we present the newly developed software PORT that implements the local processing part of this workflow, protecting privacy by shielding sensitive data from outside observers, including the researchers themselves. When using PORT, researchers can tailor the local processing procedure suitable to the data download package and research question. Thus, PORT enables a host of potential applications of social data science to hitherto unobtainable data.

## Highlights

• Software that allows for privacy-preserving analysis of digital trace data
• Participants can give true informed consent regarding data they share with researchers
• The software is provided via open source
• The software can be tailored toward different research questions or data sources

## The bigger picture

Since the General Data Protection Regulation, individuals can request a copy of all the digital traces they leave behind, which are then provided in so-called data download packages (DDPs). This makes it theoretically possible for individuals to share these DDPs for research purposes. However, DDPs can contain very sensitive information, making individuals unwilling to share them with researchers. In addition, researchers are often interested in only a small part of the large amount of information that is found in the DDP.

The software introduced in this paper overcomes this privacy issue that currently prevents the use of DDPs for scientific research. By doing so, the huge amount of digital traces that are left behind by individuals in many aspects of their lives are finally becoming available for research purposes, while the participants are involved in the sharing process and can provide true informed consent regarding the information that they share.

## Graphical abstract

**Figure:**
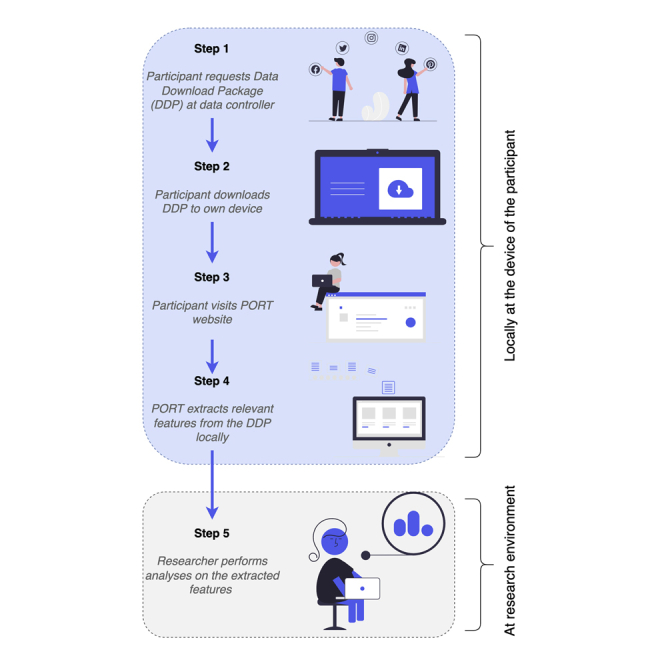

## Teaser

The software introduced in this paper allows for a privacy-preserving analysis of digital traces by researchers. As individuals generate digital traces in more and more aspects of their lives, this software can open up new sources of data to researchers from various disciplines. For example, it can provide access to social media data for social and behavioral scientists, open up bank transaction or energy use data to economists, or release electronic health records to health scientists.

## Introduction

The General Data Protection Regulation (GDPR) grants all natural persons within the European Union (EU) the right to an electronic copy of their personal data as collected by data controllers upon request. All major data controllers, such as social media platforms, banks, online shops, loyalty card systems, and public transportation cards comply with this right by providing their clients with a “data download package” (DDP). However, as only data subjects have the right to receive these DDPs, these digital traces cannot currently be accessed or used for scientific research. DDPs have many potential advantages. For example, they constitute behavioral data that can measure phenomena the participant may not easily remember. Furthermore, DDPs often contain retrospective data that allow the researcher to go back in history, well before the date of the study, to look at the participant’s behavior. Furthermore, the data donation approach can easily be embedded within a larger survey, facilitating the sample selectivity corrections that are necessary for digital trace data (e.g., Olteanu et al.^1^). For a more in-depth discussion of both the advantages and further issues relating to DDP data donation, please see Boeschoten et al.^2^

Recently, a workflow was introduced that allows researchers to analyze the digital traces found in DDPs while preserving the privacy of research participants.^2^ Here, a participant downloads the DDP onto their personal device. Next, a local processing step extracts only the features relevant for the research project from the DDP. After inspection and informed consent by the participant, these extracted features are sent to the researcher to perform the analysis of interest.

In this paper, we introduce a proof-of-concept of software that enables the local extraction step of this workflow. This step takes place locally at the participant’s device in a web browser, so researchers only need to share a URL with participants. Table 1 gives an overview of important definitions related to this workflow. The aim of this paper is to illustrate that the previously introduced workflow is not only a theoretical framework but can also be applied in practice. In addition, developing and applying our software PORT gave us more concrete insight into the ethical and practical considerations at play when using PORT. Therefore, we aim to present these along with our recommendations, and discuss challenges that require more research.

**Table 1:** Overview and explanation of the key actors when digital trace data are donated for research

General Data Protection Regulation (GDPR)	A legislation in the EU that (among other things) grants natural persons the right to receive their personal data from data controllers in a machine-readable format
Data controller	An entity, such as a tech company or public authority, that collects data on natural persons and is therefore required by the GDPR to provide that data in a DDP to the participant
Data download package (DDP)	A machine-readable electronic file containing the personal data requested from the data controller by the participant
Participant	A person who is asked by the researcher to donate data from their DDP to the study
Researcher	A scientist who needs participant data encased in DDPs to answer a (social-scientific) research question
Data donation	An individual’s active consent to provide their personal data for research purposes (Skatova and Goulding^3^)

Previously, there have been other initiatives for software that extracts information from DDPs. For example WebHistorian by Menchen-Trevino^4^ allows individuals to visualize different parts of their Google DDP, and has, for example, been applied by Wojcieszak et al.^5^ to investigate how internet users arrive at certain sources of news. Alternatively, Araujo et al.^6^ developed OSD2F, which does not allow the local processing to completely take place before uploading and donating but does allow participants to inspect their data in order to let them decide which parts to share. The software introduced in this paper is unique in the sense that it allows the researcher to choose which DDP is used, which data formats are processed, which types of features are extracted, and how the data are summarized. These aspects differ from project to project. The software allows the researcher to develop a Python script specifically for their research question. The software can thus be easily adapted to the specific research question under investigation. Although the software allows us to summarize the features in such a way that no Person Identifiable Information (PII) is shared, it is the responsibility of the researchers to continue to meet this condition once they develop their own extraction script. It therefore allows greater privacy protections for respondents while remaining flexible enough for research purposes.

In the next section, we provide a recap of the recently introduced workflow for data donation. Next, we describe how the software works and discuss its technical specifications. Third, we discuss plans for future work and guidelines for ethical use. Fourth, we illustrate the use of the software with two applications, followed by a conclusion and discussion.

## Results and discussion

### Methods

In this section, we first describe step by step the procedure as perceived by the participant. We then describe in more detail the software that has been used to enable this procedure. We have named the software “PORT,” as it serves as a “port” through which data are transferred from participants to researchers.

#### Workflow

When a participant is invited for a research project that uses PORT, the first step is always that the participant requests the DDP in which the researcher is interested from the respective data controller. It is the responsibility of the researcher to provide the participant with a clear, detailed, and accurate description of how the DDP can be requested, although many of the larger data controller companies already provide these descriptions on their websites (Facebook; WhatsApp; Twitter; Snapchat; Instagram). The amount of time it takes for the participant to receive the requested DDP varies between a couple of seconds and up to weeks, depending on the size of the DDP and the extent to which the data controller has automated the process.

In the second step, the participant downloads the DDP to their own device. The way the participant receives the DDP can differ per the data controller. In some cases, a file can be downloaded directly and stored locally on the device of the participant. At the time of writing, many banks, online stores, energy providers, and public transport companies provide this option. However, in most cases the preparation of the DDP by the data controller takes some time and the participants receive an e-mail or a notification within the platform of the data controller containing a download link. Google provides an option to place the latest version of the DDP on the participant’s Google Drive and allows the user to update the DDP automatically.

After the DDP is downloaded and stored, the third step is for the participant to visit the researcher’s project website at PORT.

In step 4, PORT extracts relevant information from the DDP; it does so locally and without communication with a remote server. In the next subsection, more detail regarding this procedure is provided. Note that up until this moment no information is shared with the researcher. Once PORT’s extraction algorithm is finished, it displays the extracted information and requests consent from the participant to share this with the researchers. If the participant consents, the extracted information is then encrypted and sent to the researcher for analysis.

Once the researcher has obtained the extracted data of all participants, analyses can be performed to answer the research questions of interest in the fifth step.

#### Software

**Figure 1:**
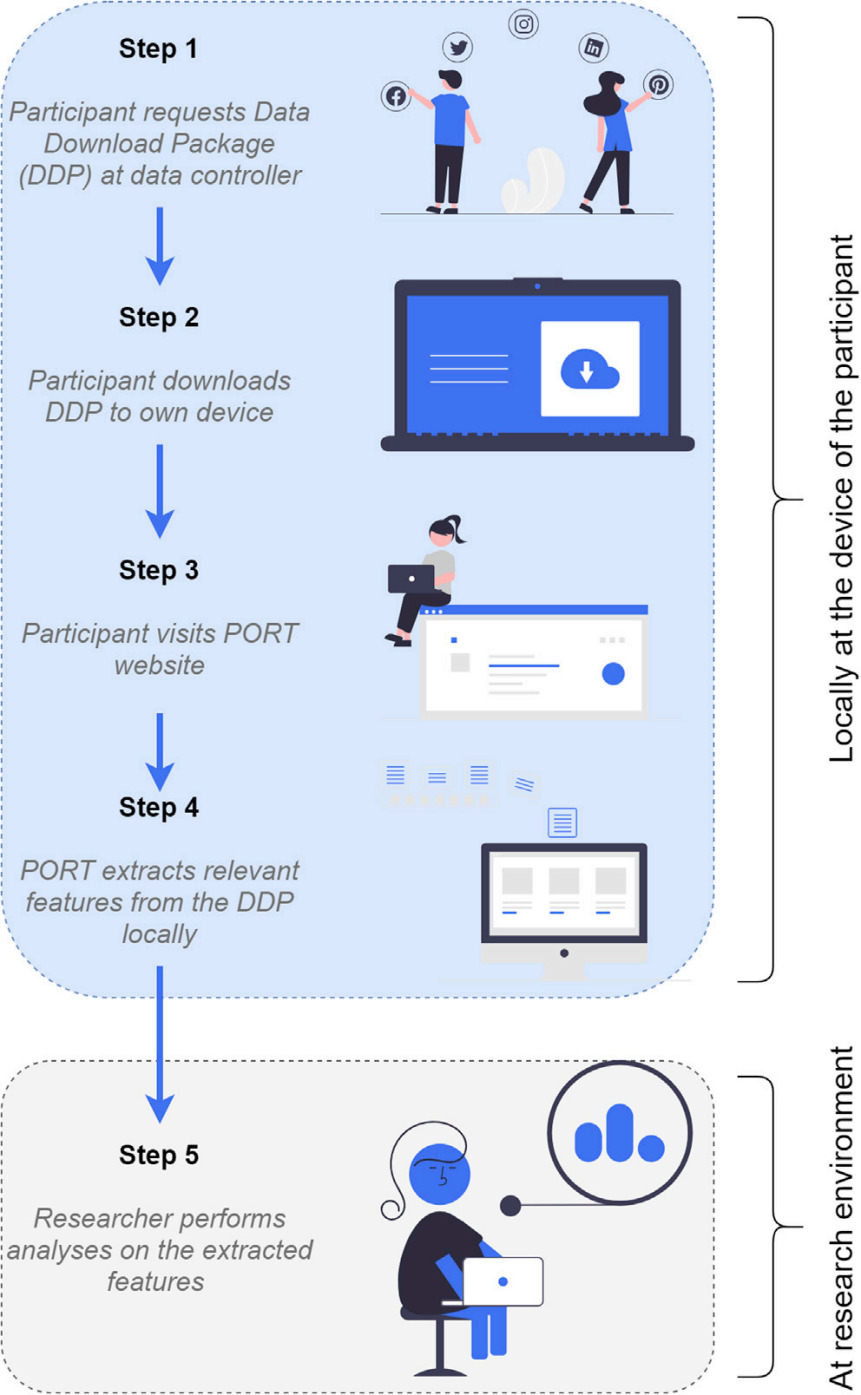
Step-by-step illustration of the workflow that allows for a privacy-preserving analysis of data download package, locally on the device of a research participant

In the workflow, the extraction of relevant features of the digital trace data (the fourth step in Figure 1) is crucial. We ensured that this step takes place locally at the device of the participant, thereby preserving the privacy of the participant, by building PORT. PORT is a WebAssembly application that can run within browsers on both PCs and mobile devices.

**Figure 2:**
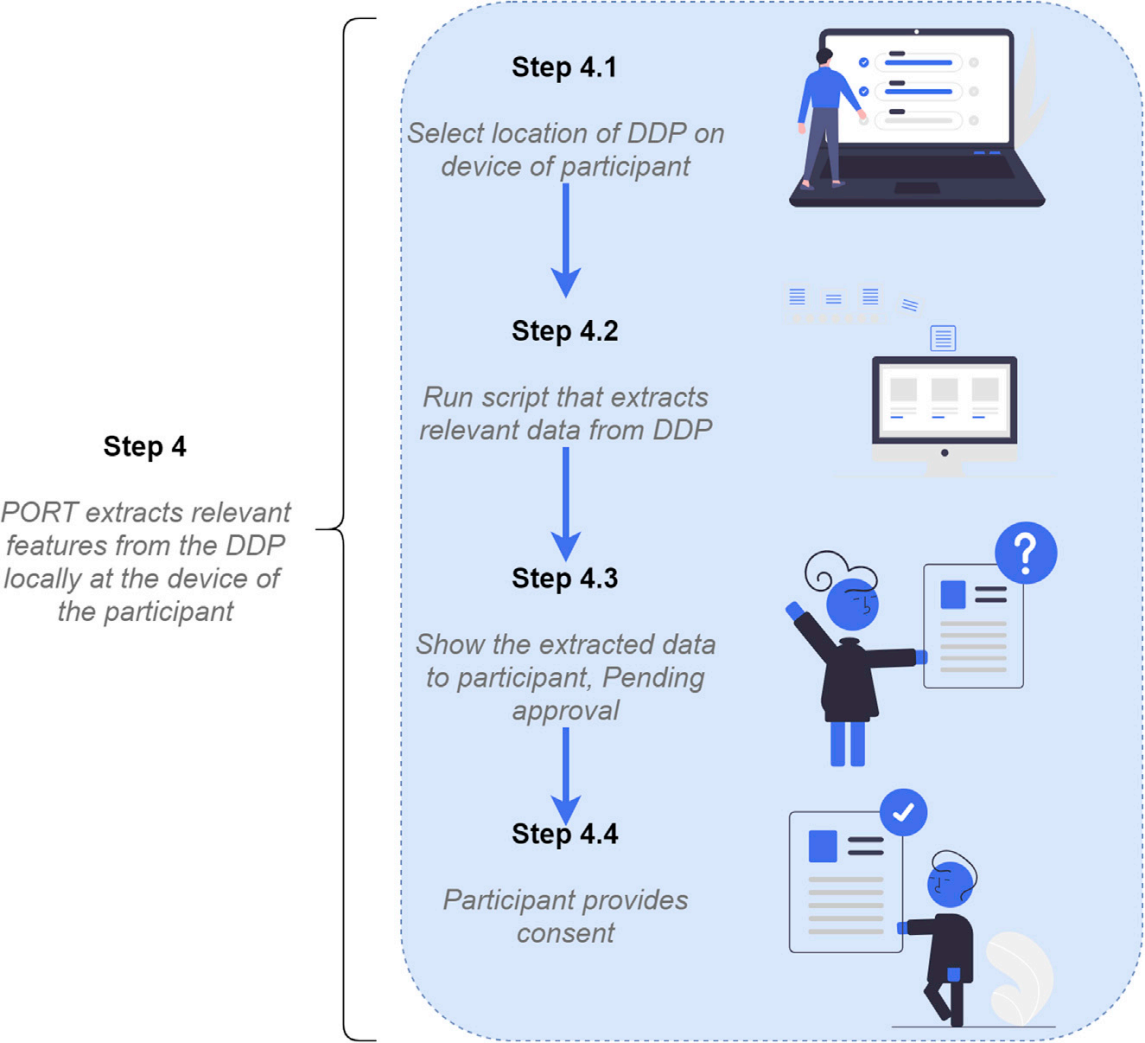
A more detailed illustration of the steps taken by a research participant during the local processing phase of the proposed workflow, which can be performed using the PORT software

Figure 2 provides more detail regarding PORT’s functionality. First (step 4.1), the participant selects the location in which the DDP is stored at the device. Once the DDP is selected, PORT runs a script in the browser that locally extracts the data from the DDP that is relevant for the researcher (step 4.2). The data extraction script consists of code written by the researcher in Python that is tailored to the specific research question and the specific DDP.

PORT makes use of Pyodide,^7^ an open-source library that enables running Python in a web browser through having the Python interpreter compiled to WebAssembly.^8^ WebAssembly is an open standard for portable binary-code format, enabling high-performance applications on the web. Thus, the custom Python scripts will run in a safe, sandboxed environment while the browser’s security and permission policies are enforced. When the participant clicks the “process” button in PORT, the DDP is sent to the WebWorker. The JavaScript code then presents the DDP through the Pyodide bridge application programming interface as a regular Python file object. A function in the Python code is invoked, which receives this file object and interacts with it.

Once the function has extracted the relevant data, it returns the extracted data accompanied by information about what exactly has been extracted. These data are then converted by the Pyodide bridge to JavaScript object types. The JavaScript within the WebWorker creates an event in Java and notifies the primary JavaScript. This JavaScript code then updates the browser domain^9^ to present the extracted data to the participant (step 4.3). Conceptually, this step can be considered as if a small system environment is built within the browser, completely separated from the device on which it is run. This is a safe procedure, as the environment is able to process the data uploaded in the environment but is not able to access anything else that is present on the device. In addition, this system environment is destroyed as soon as the browser page closes.

In the final step (step 4.4), the participant inspects the extracted data and can provide informed consent for sharing these data with the researchers by clicking the respective button. If clicked, the extracted data are shared with the researchers over the internet; note that this is the first communication of the participant’s device with a server controlled by the researchers. Alternatively, this moment can also be used to decline consent. PORT currently uses an Elixir, Phoenix server hosted by Eyra Leap (Den Haag, the Netherlands). For future use, integration with preferred online repositories or servers hosted by academic institutions is also possible, allowing for the extracted data to be sent directly to the location of interest.

### Future work and guidelines for ethical use

Although we currently only present a proof-of-concept of PORT, we provide a first set of guidelines to help researchers to use PORT legally and ethically. Our considerations are discussed for: (1) the participant recruitment phase; (2) the local processing phase; and (3) the data extraction phase.

The first contact between the participant and the researcher is made at the participant recruitment phase. Here, a researcher might use a professional participant recruitment platform or an existing panel study. Regardless of the exact structure, it is important to keep in mind that the researcher is responsible for generating unique identifying keys for each participant, which can be used in such a way that once the extracted features are stored they are labeled with this key and can, for example, be used for linkage to survey measures or to make conclusions regarding the selectivity of the final obtained sample. At this stage, it is good practice to inform participants regarding the study aims, exactly the type of data they will share, and the process they will go through. The sample of participants who are willing to participate, able to go through the complete process, and have the DDP of interest is probably highly selective when compared with the target population. Therefore, researchers have to rely on correction approaches such as weighting or (multiple) imputation. These correction procedures can benefit from supplementing the data donation procedure with survey questions. More detail on such issues and guidelines for further use can be found in Boeschoten et al.^2^

During this participant recruitment phase, there are two important considerations in terms of data security. First, the researcher should ensure that the unique identification keys are anonymous and that linkage takes place in a secured environment. Second, the researcher should provide clear instructions that the DDPs should be stored on the participant’s personal device and not in the cloud or a device owned by someone else. In addition, even if the DDP is stored at the participant’s own device, there is always a small risk that this device is, for example, stolen or hacked. Therefore, the most straightforward option to minimize risks is to make participants aware of their data still being present on their device after they have completed the donation process and to suggest that they delete such data.

For the local processing phase, the researcher prepares a Python script. This script should adhere to a number of rules. First, it extracts only the features from the DDP that are relevant for the particular research question. Note that if these extracted features are sensitive, the collected data should be treated as such in the subsequent steps, e.g., the safety measures taken at the data storage facility. Second, the script handles variability in structure and content over DDPs from different participants. Third, the script presents the extracted features in a clear and intuitive way for the participant to review when providing informed consent and for the researcher to process for further analysis, including linkage to the data from other participants or linkage of other data sources regarding the same participant, for example survey data. Regarding the presentation of extracted features to the participant, the researcher can present the features in an intuitive way, using a table, a graph, or a figure such as a map. However, all features shared with the researcher should have been presented to the participant.

During this local processing phase, there are two security issues, the first of which lies in the Python script itself and is described above. If for one of these reasons sensitive data are extracted from the packages, the extracted data should be treated as such in the remainder of the process. Second, the local processing software, PORT, runs in the web browser of the participant. Security in the sense that no third parties are accessing these data is thereby also dependent on the security system embedded in this web browser. For the last step, during the data extraction phase, participants should therefore be encouraged to use a trustworthy browser.

When using PORT, we encourage researchers to adhere to the FAIR principles^10^ while retaining the preservation of privacy if the type of features extracted demands this. To encourage findability of the research projects using PORT, a separate project page is available on the website of PORT for each project, which can be accessed from the starting page. At the project page, information about researchers and research findings can be shared, which can be updated throughout the duration of the project. Furthermore, the Python script used to extract features is made publicly available here so that it can be reused for other projects. Lastly, information regarding the location where the data will be stored should be provided.

Accessibility of the extracted features very much depends on the sensitivity of its content, and different options are possible. Researchers can for example choose to let the collected data be collected at the Eyra server or to let the extracted features be sent directly to a repository in a protected (cloud) environment. As the nature of PORT is interoperable, it can be combined with different participant recruitment and data storage platforms. However, whether a particular platform can be combined in practice depends on platform-specific characteristics. PORT is developed open source and we ask users to share their developed Python scripts open source as well, such that they can be reused. Furthermore, once researchers have inspected and cleaned the collected data where needed, they can provide information for other researchers regarding if and how their collected data can be reused for other studies.

During this data extraction phase, the security of the extracted data depends on three aspects. First, during the process of sending the data to the server, the data should be encrypted to ensure other parties cannot obtain access to the data during this process. Second, extraction depends on the security of the server to which the data are sent. Third, the security depends on how the researchers handle the data afterward. These last two steps are similar for any other type of sensitive research data.

### Example applications

In this section, we illustrate how PORT can be used to extract features from DDPs to collect digital trace data for answering research questions. We provide two applications in which we select a suitable DDP and develop a Python script that extracts the information needed from the DDP. We initially developed the script by inspecting our personal DDPs. Based on the data structures found here, we simulated a DDP. The simulated DDP is then used to illustrate how PORT is able to locally extract features from it. Next, PORT shows the extracted features, after which informed consent can be provided. Because we used simulated DDPs, the steps in the applications are reproducible.

#### Measuring differences in where time is spent during Covid-19 lockdowns

In this example we focus on the research question “how does travel behavior change in times of a Covid-19 lockdown?” Since self-reporting instruments such as time-use surveys or diaries are prone to recall bias,^11^ digital trace data are an interesting alternative. DDPs are particularly relevant here, as the researcher is interested in retrospective data. Such past data are difficult to obtain using, for example, wearables: the researcher has to anticipate that “something interesting” will happen in the future. In contrast, DDPs that provide location data generally do so for a considerable portion of the past.

When exploring different data controllers that store location information, we focus on DDPs collected by the Android operating system, as Android has the majority market share in Europe.^12^ Researchers can consider to also collect DDPs that store location information via, e.g., iOS, to reduce the amount of missing data induced due to the participants not having an Android DDP. In the variety of DDPs collected by Android and Google (which can be found under “Google Takeout”), the “Google Semantic Location History” (GSLH) contains monthly .JSON files with information on geolocations, addresses, time spent at locations, activity, and more.

#### Google Semantic Location History data extraction

Information on time spent at locations and traveled distances is easily extracted from the GSLH DDP, as it contains start and end time of each visited place and activity as well as the distance traveled per activity. We simply need to select and sum the appropriate values per month. Note that the DDP contains time information in milliseconds since January 1, 1970, and distance information in meters. For readability, we convert these to days and kilometers, respectively. Figure 5 shows 49, 47, and 19 visited places for 2019, 2020, and 2021, respectively.

##### Donating GSLH data using PORT

**Figure 3:**
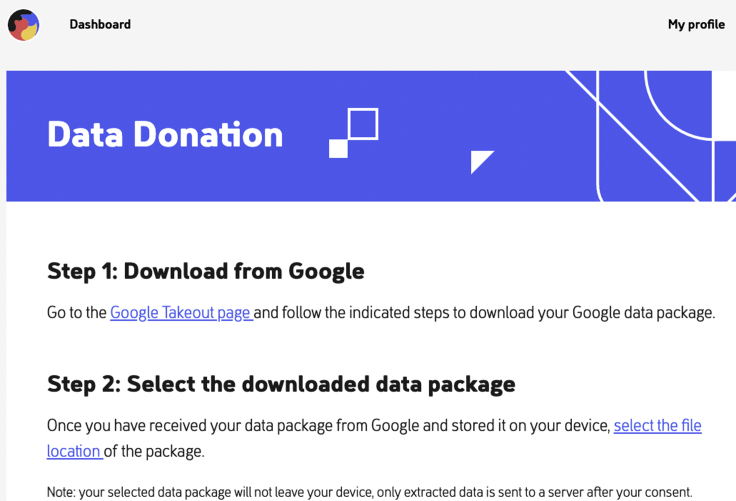
Once the participant has downloaded their GSLH or other DDP, they can select the file location on their device for data donation using PORT

When participants are invited to participate in this study, the first step is that they are requested to download their GSLH DDP, as shown in Figure 3.

**Figure 4:**
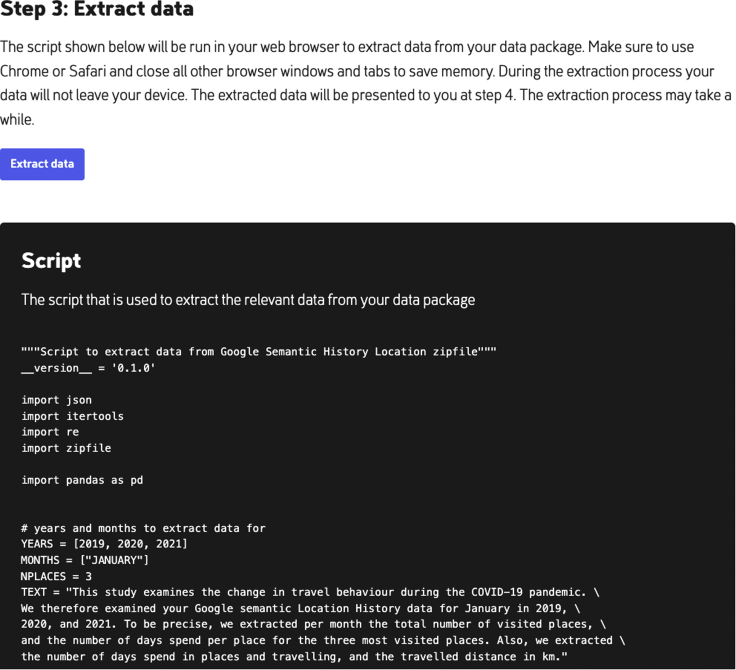
With the “process data package” button in PORT, the GSLH Python extraction script extracts the relevant features from the selected DDP For transparency, PORT shows the complete Python script.

Once the GSLH DDP is downloaded and stored, the participant can click on the “select the file location” button at PORT as seen in Figure 3. Once the DDP is uploaded in PORT, the third step becomes visible on screen, as seen in Figure 4. At this step, for transparency the extraction script is visible for inspection. Once the participant clicks the “process data package” button, the extraction script is run on the selected DDP.

**Figure 5:**
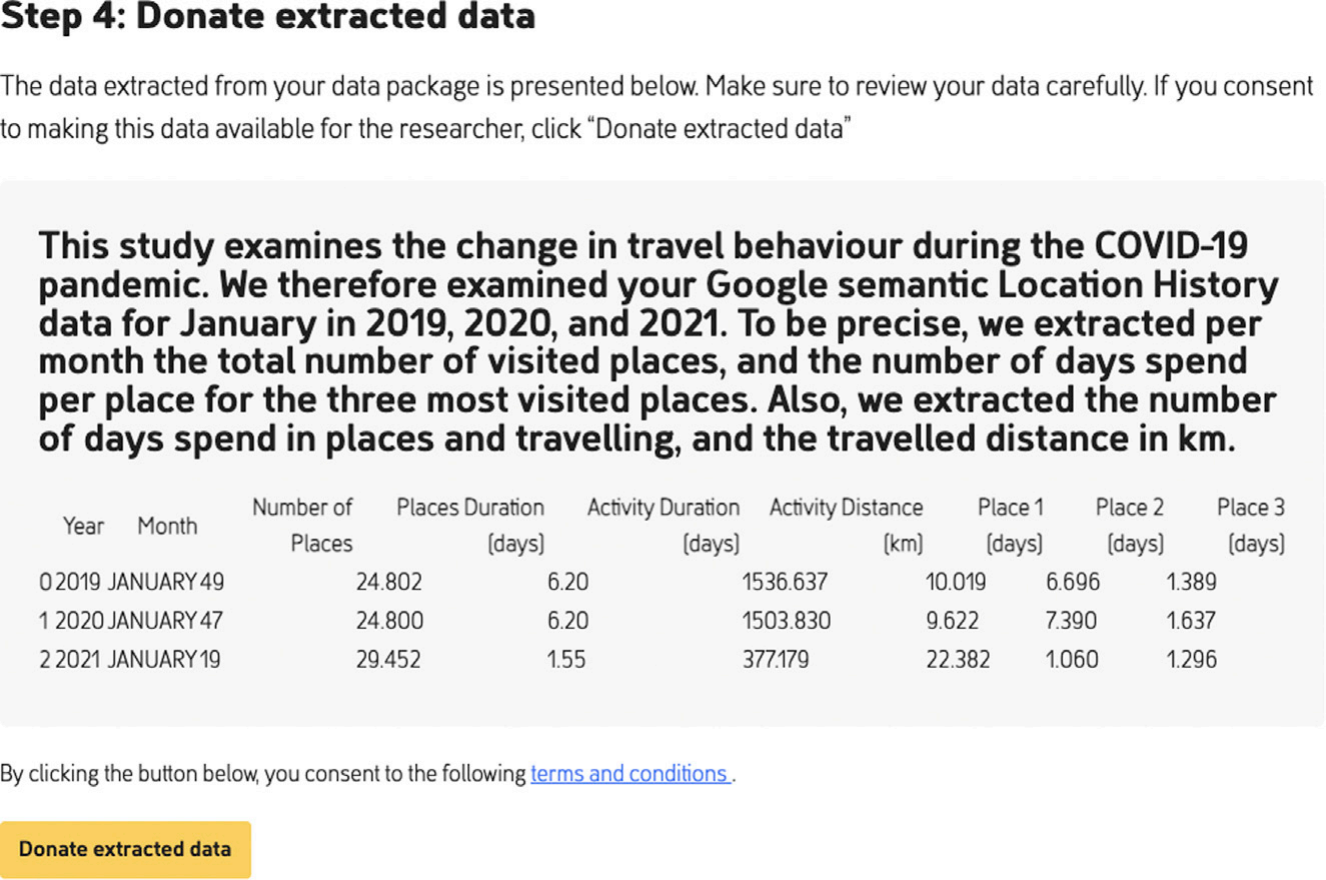
Extracted data from the Google Semantic Location History DDP as displayed to the participant Below the extracted data, the participant can find the “donate extracted data” button.

Once the script is finished, the extracted information is shown to the participant, including an explanation of what the various printed numbers exactly mean, as shown in Figure 5. Note that the extracted data here contain no PII. Below the extracted information there is a button, “donate extracted data.” When the participant clicks this button, the extracted data are transmitted to the server of the researcher to which PORT is connected, so the complete DDP is never shared with the researchers.

#### Measuring differences in online news consumption due to the Covid-19-induced curfew

The second application focuses on the research question “how does news consumption change during times of a Covid-19 curfew?,” which is closely related to the research conducted by Broersma and Swart^13^ on changes of news habits during the Covid-19 pandemic. Google Chrome is the most frequently used browser, with a 65% market share.^14^ Therefore, it makes sense to again obtain the information that can help to answer this research question via the DDP of Google, Google Takeout. Within this DDP, the browsing history of Google Chrome is found in the file “BrowserHistory.JSON.” This file contains a user’s entire compiled browser history, from the moment they started using Google Chrome or since the last time they deleted their browsing history.

##### Google search history data extraction

Similar to the previous application, the DDP was simulated. More details can be found in Appendix B. Analyzing the search behavior as listed in the BrowserHistory.JSON file consists of a number of steps. First, the BrowserHistory.JSON file is extracted from the provided Google “Takeout.zip” file, and is loaded as a dictionary. Using the timestamp provided for every search in the BrowserHistory.JSON file (*time_usec*), it can be determined in what period the website was visited, i.e., before the start of the curfew, during the curfew, or after the curfew.

Second, it is determined whether a certain website is a news site or not, and for both groups the number of individual visits are counted. To determine whether a website is a news site or not, each website visit’s URL is matched with a list of the most popular Dutch news websites according to Wikipedia.^15^ In addition, for each website visit, it is determined whether the visit took place before, during, or after curfew, and during morning, afternoon, evening, or night, based on the supplemented timestamp. Finally, all information is combined into a single table, where each row represents a different profile (e.g., news sites/before curfew/morning) and sums the total number of individual website visits corresponding to that profile (see also Figure 7). When this information is collected from a group of participants, a researcher can determine whether news consumption increases during a period of curfew, especially in the evening and at night, compared with periods without a curfew.

##### Donating Google search history data using PORT

**Figure 6:**
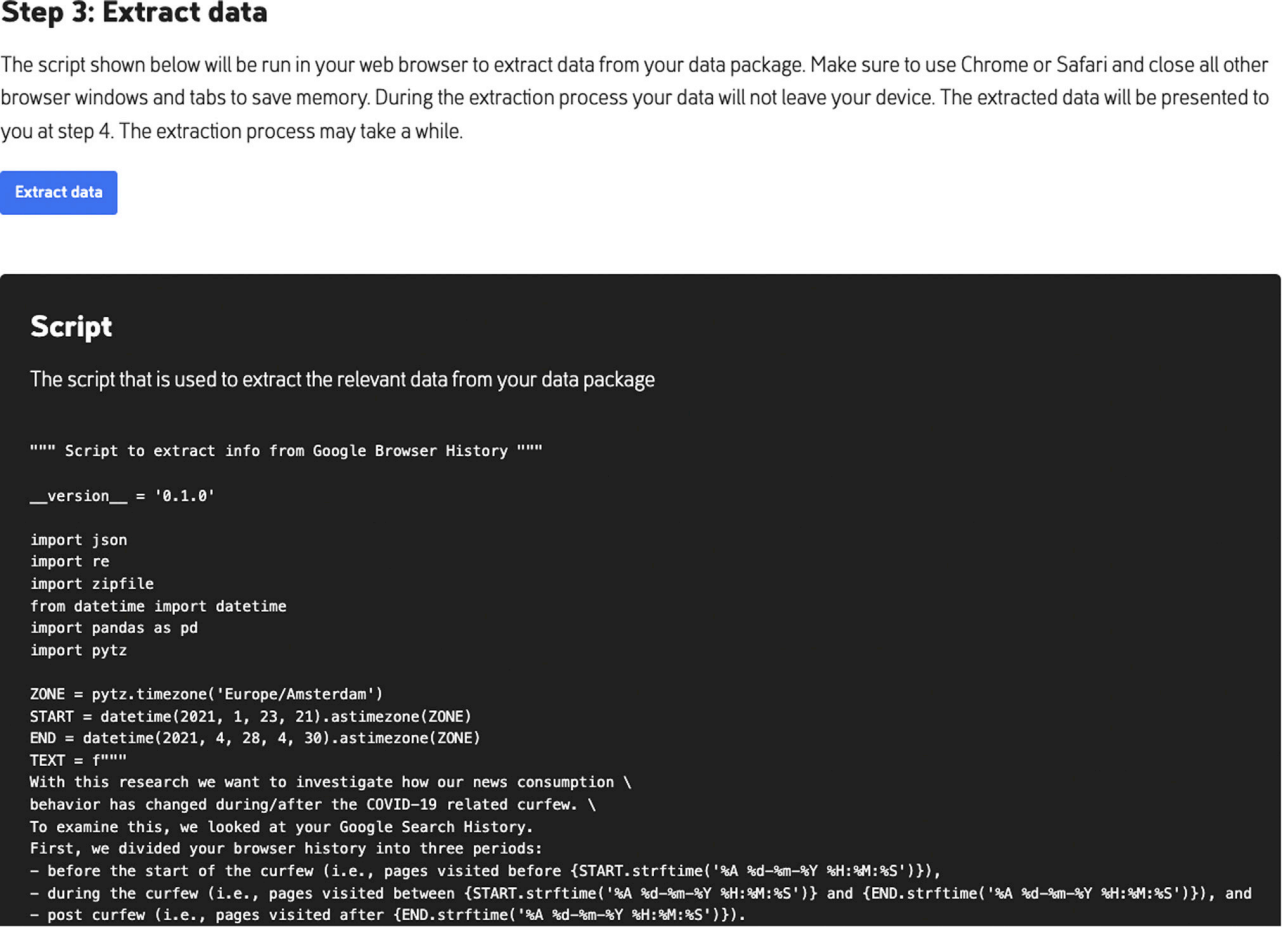
With the “process data package” button in PORT, the Browser History Python extraction script extracts the relevant features from the selected DDP For transparency, PORT shows the complete Python script.

**Figure 7:**
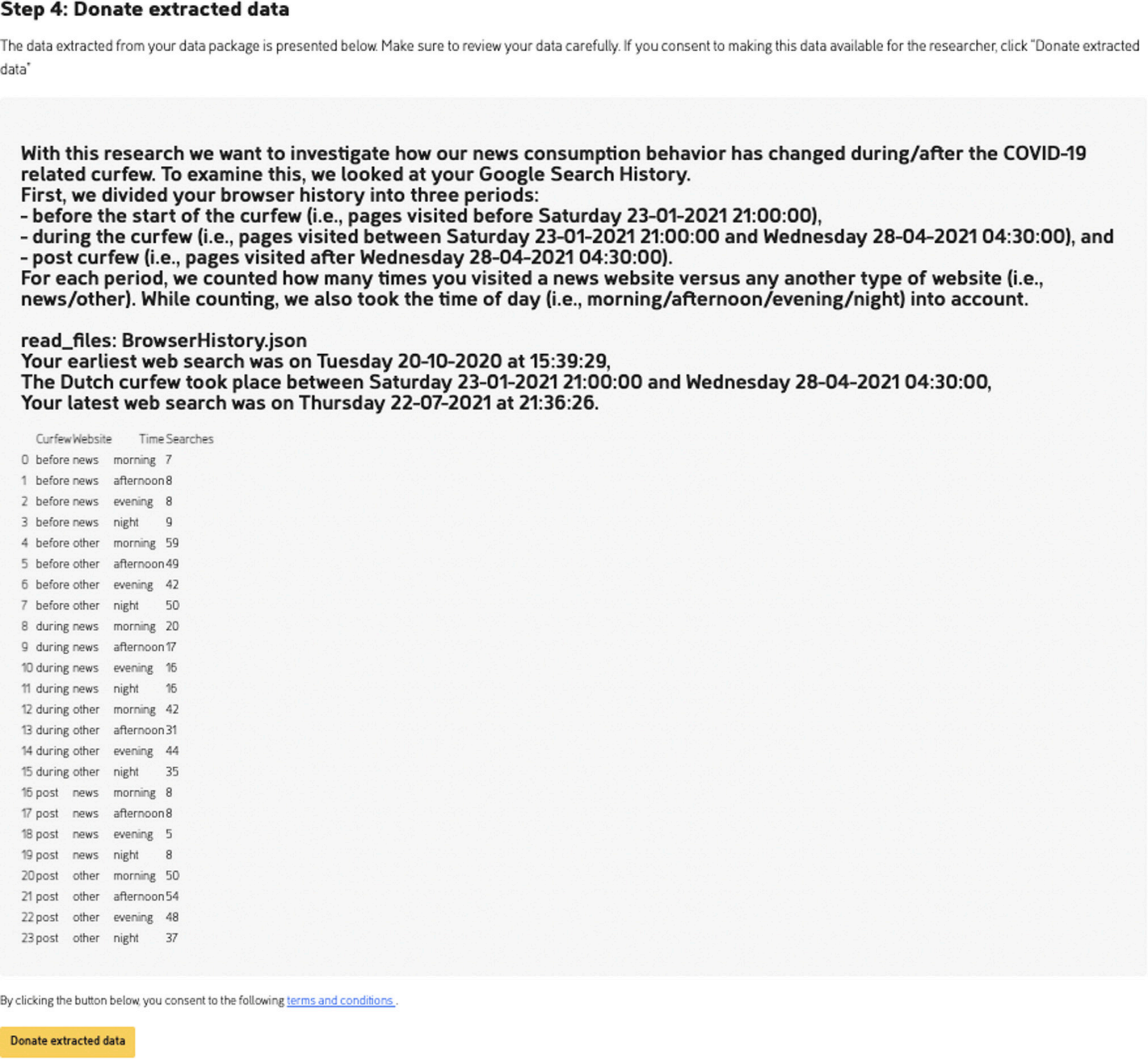
Extracted data from the Browser History DDP as displayed to the participant Below the extracted data, the participant can find the “donate extracted data” button.

Similar to the first application, participants start by requesting their personal DDP via their Google profile. Again, the participant uploads the DDP in PORT for the package to be processed (Figure 6), after which the extracted data are shown (Figure 7). Again, the extracted data contain no PII. Instead, a frequency table of the amount of (news) sites that were visited at different periods is shown.

### Conclusion

This paper introduces a proof-of-concept of software that locally, on a participant’s device, extracts relevant features from DDPs. Thanks to this software, researchers can now use digital trace data for research purposes while preserving the privacy of participants and with their informed consent.

To use PORT in practice, it should be integrated with a participant recruitment platform or an existing panel study such as LISS^16^ or Understanding Society.^17^ In addition, it should be integrated with a data repository such as DataVerse^18^ or Surfdrive.^19^ At the moment of writing these options have not yet been developed, although we are working on it for our first application studies. However, as all the code is available open source, researchers are free to use it and integrate it with their own participant recruitment platform and servers.

When using the workflow as proposed by Boeschoten et al.^2^ and PORT, the only task that remains is for data scientists and applied researchers to collaborate to develop a high-quality extraction script in Python that is flexible in terms of handling a variety of data structures (see Boeschoten et al.^20^). When developing such an extraction script, it is important to find a balance between ensuring on the one hand that all information relevant for answering the research question of interest is extracted, while on the other hand no sensitive data are unnecessarily collected.

Besides discussing how PORT can be used in practice, it is also important to discuss the type of research questions for which PORT is less suitable or which pose challenges to its use. A first example considers research questions of a more exploratory nature. For such questions, an extraction script might not be suitable. However, even in such situations a researcher can consider using the workflow and software but, instead of using an extraction script, a de-identification script might be more appropriate. An example of such a de-identification script has been developed by Boeschoten et al.^20^ for Instagram DDPs, which only selects the files within the DDP that are of interest to the research and then removes all identifiers from these files. When applying the workflow in such a way, two main study principles are still applied: the privacy of research participants is protected and only the necessary data are collected. A second example of situations where PORT might be less suitable is research questions that consider participants who are less proficient in the use of modern technology. In general, we aim to focus more on the intuitiveness of the workflow and ways to engage participants in future research. Engaging these less proficient participants will likely also require substantive investments in assistance throughout the process. However, it might be the case that for these participants less information can be found in DDPs and, therefore, the approach of using donation of data extracted from DDPs is less useful in general. A third example of situations where PORT might be less suitable relates to research questions for which balanced samples are of utmost importance. Ohme et al.^21^ illustrate that besides proficiency in the use of modern technology, privacy concerns also affect the willingness to participate in data donation studies. In general, depending on the topic of the study, combining data collected through data donation with data regarding the same participants collected by other means, such as surveys or administrative registers, can help to provide insight into the extent of these issues, i.e., in terms of missing data mechanisms, whether the missingness due to privacy or technology concerns are data missing at random or missing not at random.^22^ In general, integrations with existing panels and extensions allow for more DDPs to be collected or multiple algorithms to be run and can also help users to provide insight into the quality of their collected data. Boeschoten et al.^2^ provide more detailed suggestions along these lines.

To conclude, PORT opens up vast new research opportunities for researchers with an interest in digital trace data. Digital trace data can be collected in DDPs by a substantive part of the world’s population and in regard of every aspect of their (digital) lives, such as social media, banks, online shops and shops with loyalty card systems, travel and movement behavior, and health. PORT allows for a privacy-preserving analysis of these digital traces for research purposes, subject to true informed consent.

## Experimental procedures

### Resource availability

#### Lead contact

Requests for reagents and resources can be directed to and will be fulfilled by the lead contact, Laura Boeschoten (l.boeschoten@uu.nl).

#### Materials availability

This study did not generate new unique reagents.

## Data Availability

All original code has been deposited at Zenodo under https://doi.org/10.5281/zenodo.5703454 and is publicly available as of the date of publication.

## Appendix A: Google Semantic Location History data simulation

The code used to generate the simulated GSLH DDP builds on the open-source Python packages “GenSON,”^23^ “Faker,”^24^ and “Faker-Schema.”^25^ GenSON is used to generate a .JSON schema of a personal DDP. The .JSON schema describes the format of the GSLH data, and can be used to generate dummy data with a similar format. Dummy data are then generated by converting the .JSON schema to a custom schema expected by faker-schema in the form of a dictionary, where the keys are field names and the values are the types of the fields. The values represent available data types in Faker, packed in so-called providers. Faker provides a wide variety of data types via providers, for example for names, addresses, and geographical data. This allows us to easily customize the dummy data to our specifications.

We generated GSLH dummy data for the years 2019, 2020, and 2021. First we created 50 dummy addresses with Faker, with coordinates in the Netherlands within a radius of 0.1° of each other. For the years 2019 and 2020, the visited locations were randomly chosen with weighted probabilities from all 50 addresses; for 2021 only 20 of these addresses were available for random selection. The weighted probabilities were used to create a top three of most frequently visited locations. We also varied the duration of time spent at locations per year, whereby in 2019 and 2020 80% of the time is spent at locations and in 2021 95% of the time is spent at locations. The geodesic distances between visited locations were calculated in kilometers using “GeoPy.”^26^

## Appendix B: Google search history data simulation

The BrowserHistory.JSON file is structured in such a way that, for each web search, information about that specific web search is listed in a dictionary. Such a dictionary entails information about how the website was visited (*page_transition*, i.e., “LINK,” “GENERATED,” or “RELOAD”), the title of the web page (*title*), the complete URL to the weblink (e.g., https://nos.nl/), a unique identifier for the user (*client_id*), and the timestamp of the visit (*time_usec*). As the BrowserHistory.JSON file is a mere compilation of all these unique search dictionaries stored within one overarching “Browser History” dictionary, the code used to generate the simulated data is quite straightforward.

First, an empty dictionary is created with the aforementioned structure. For each simulated web visit, a random *page_transition* (i.e., “LINK,” “GENERATED,” or “RELOAD”) is chosen using the “Random” package in Python,^27^ which uses the Mersenne Twister algorithm.^28^ Second, the *title* and *url* of the web page are constructed using the sentence( ) and website( ) functions of the open-source Python packages “faker”^24^ and “random,”^27^ respectively. After the URLs are created, the homepage part of the URL is replaced with a randomly selected Dutch news website for n% of the URL. These news sites are randomly picked from a sampled list of popular Dutch news websites as obtained from Wikipedia.^15^ The *client_id* is created using a random set of letters and digits, making up a string of length 10. Finally, timestamps are generated in such a way that morning, afternoon, evening, and night times occur evenly.

Note that the simulated browser history data are created in such a way that there is a difference in total number of news versus random website visits before (before 2021-01-23), during (between 2021-01-23 and 2021-04-28), and after (after 2021-04-28) the curfew. The default settings will result in a “Takeout.zip” file, with a sub-folder “Chrome” leading to a “BrowserHistory.JSON” file, i.e., a Browser History dictionary containing 1,000 web searches. During the period of the curfew—compared with the period before or after the curfew—there were 15% more news web visits and, overall, more web visits during the evening.
